# Instrumented Timed Up and Go Analysis Identifies Biomechanical Markers Across Early Hoehn and Yahr Stages of Parkinson’s Disease

**DOI:** 10.3390/s26165177

**Published:** 2026-08-15

**Authors:** Paula Molero-Mateo, Carlota Trigo, Adriana Torres-Pardo, Diego Fernández-Vázquez, Diego Torricelli, İrem Akgün, Jorge Andrés Gómez-García, Marina Algaba-Vidoy, María Carratalá-Tejada, Simón García-Diego-Martínez, Víctor Navarro-López, Yeray González-Zamorano, Isabel Mª Alguacil-Diego, Francisco Molina-Rueda

**Affiliations:** 1 Escuela Internacional de Doctorado, Rey Juan Carlos University, 28933 Móstoles, Spain; paula.molero@urjc.es (P.M.-M.); simon.garciadiego@urjc.es (S.G.-D.-M.); 2 Laboratory of Movement Analysis, Biomechanics, Ergonomics and Motor Control (LAMBECOM), Faculty of Health Sciences, Rey Juan Carlos University, 28922 Alcorcón, Spain; diego.fernandez@urjc.es (D.F.-V.); maria.carratala@urjc.es (M.C.-T.); victor.navarro@urjc.es (V.N.-L.); isabel.alguacil@urjc.es (I.M.A.-D.); 3 Department of Physical Therapy, Occupational Therapy, Physical Medicine, and Rehabilitation, Faculty of Health Sciences, Rey Juan Carlos University, 28922 Alcorcón, Spain; yeray.gonzalez@urjc.es; 4 BioRobotics Group, Centre for Automation and Robotics (CAR), Spanish National Research Council, 28006 Arganda del Rey, Spain; carlota.trigo@csic.es (C.T.); adriana.torres@csic.es (A.T.-P.); diego.torricelli@csic.es (D.T.); jorge.gomez.garcia@csic.es (J.A.G.-G.); marina.algaba@csic.es (M.A.-V.); 5 E.T.S. Ingenieros de Telecomunicación, Universidad Politécnica de Madrid, 28040 Madrid, Spain; 6 Department of Physiotherapy and Rehabilitation, Faculty of Health Sciences, Gaziantep University, 27410 Gaziantep, Türkiye; iremakgun@gantep.edu.tr; 7 Cognitive Neuroscience, Pain and Rehabilitation Research Group (NECODOR), Faculty of Health Sciences, Rey Juan Carlos University, 28922 Alcorcón, Spain

**Keywords:** Parkinson’s disease, Instrumented Timed Up and Go, biomechanical markers, gait analysis, motor control, wearable sensors, motion capture

## Abstract

**Highlights:**

**What are the main findings?**

- Turning performance was the most sensitive phase-specific marker of mobility impairment and the only parameter distinguishing healthy controls from individuals with PD at mH&Y stages 1–1.5.
- Frontal-plane control and trunk, elbow, hip, and ankle kinematics showed stage-associated differences compared with healthy controls beyond total iTUG duration, whereas gait variability remained relatively preserved.

**What are the implications of the main findings?**

- Sensor-based phase-specific mobility assessment may improve the objective characterization of functional mobility impairments in early Parkinson’s disease.
- Biomechanical metrics derived from the instrumented TUG may represent candidate digital biomarkers for mobility characterization and future disease-stratification research.

**Abstract:**

The Timed Up and Go (TUG) test is widely used to assess functional mobility in Parkinson’s disease (PD), although total test duration may overlook phase-specific biomechanical alterations. This cross-sectional study investigated whether phase-specific analysis of the instrumented TUG (iTUG) could identify candidate biomechanical markers characterizing differences among early-stage PD subgroups and healthy controls. Seventy-nine participants (38 PD, 41 controls) performed four iTUG trials in the OFF-medication state. Movement data were collected using synchronized inertial measurement units and optoelectronic motion capture systems. The iTUG was segmented into six phases, and temporal, spatiotemporal, variability, and multisegmental kinematic parameters were analyzed. Participants with PD at modified Hoehn and Yahr (mH&Y) stage 2 performed the iTUG more slowly than controls (*p* < 0.001), mainly due to impairments during walking (*p* = 0.012) and turning. Turning was the only phase that distinguished controls from individuals with PD at mH&Y stages 1–1.5 (*p* = 0.015), who also showed increased elbow asymmetry (*p* = 0.009). Participants with PD at mH&Y stage 2 exhibited broader differences, including increased double-support time, shorter stride length, lower walking speed, reduced frontal-plane control, and reduced trunk, elbow, hip, and ankle motion, whereas gait variability did not differ significantly between groups. Sagittal trunk ROM on the less affected side was the only variable that significantly differed between the two PD subgroups. These findings suggest that phase-specific iTUG analysis may reveal candidate biomechanical markers associated with mH&Y stage and functional mobility impairment and support sensor-based assessment for objective characterization of functional mobility in PD.

## 1. Introduction

Parkinson’s disease (PD) is a progressive neurodegenerative disorder characterized by impairments in motor control and intersegmental coordination, leading to altered gait patterns, reduced postural stability, and difficulties during turning [1,2]. From a biomechanical perspective, these deficits are closely related to impaired axial mobility, disrupted coordination between trunk and limb segments, and compromised dynamic balance control, ultimately increasing functional limitations and fall risk [3,4].

The Timed Up and Go (TUG) test is widely used in clinical and research settings to evaluate functional mobility in PD because it integrates multiple biomechanically demanding actions into a single, clinically feasible assessment [5,6,7]. The TUG is conventionally performed over 3 m and comprises sequential phases, including sit-to-stand, straight line walking, 180° turning, return walking, and stand-to-sit, each challenging anticipatory postural adjustments, balance regulation, and whole-body coordination [8]. Despite this complexity, TUG performance is often reduced to a single global outcome, total duration, which provides limited insight into the movement strategies used to accomplish each phase. This limitation has motivated the development of instrumented TUG (iTUG) approaches, typically based on wearable inertial sensors, enabling a more detailed and phase-specific biomechanical characterization of task performance [9,10,11].

Within this context, gait variability has been widely investigated as a key metric within instrumented assessments. Nevertheless, the literature examining this feature in early PD has reported inconsistent findings. While some authors have described increased stride-to-stride variability even in de novo patients and during OFF-medication assessments [12,13], others have reported relatively preserved gait variability during structured mobility tasks or under specific walking conditions [14]. These discrepancies may be attributed to variations in disease stage, medication status, task complexity, walking conditions, and methodological approaches [15]. Consequently, gait variability alone may not represent a universally sensitive marker of early PD, highlighting the need for more comprehensive phase-specific biomechanical assessments beyond global gait variability measures, as suggested by recent full-body optoelectronic TUG studies [16].

Accumulating evidence indicates that PD-related motor impairments are not uniformly expressed across all iTUG phases. In early and mild-to-moderate stages, overall test duration may remain relatively preserved, while specific phases, particularly walking transitions and turning, are performed using less efficient or compensatory biomechanical strategies [2,17]. Consequently, a phase-specific analysis of the iTUG may provide a more informative and mechanistic perspective, capturing underlying movement strategies and intersegmental coordination, and enabling the detection of subtle deficits that may remain masked when only total TUG duration is considered [18,19,20,21,22,23,24].

Most previous iTUG studies have evaluated participants during the ON-medication state, reflecting functional performance under routine dopaminergic treatment [5]. While this approach is clinically relevant, dopaminergic medication has been shown to improve gait speed and stride length and may also improve turning performance. However, its effects on postural control, gait variability, and axial motor function remain less consistent [15,20]. Consequently, assessments during the OFF-medication state may provide a more accurate characterization of the underlying motor phenotype by reducing the masking effects of dopaminergic therapy on subtle biomechanical impairments, particularly during turning and axial movements [22,25]. This approach is specifically relevant for the evaluation of turning and transitional tasks, as freezing of gait (FOG) occurs more frequently and with greater severity during the OFF state [26] and has therefore been adopted in biomechanical studies investigating turning performance and FOG [27].

Although previous iTUG studies have demonstrated the clinical relevance of phase-specific metrics, most have relied on a single lumbar sensor, providing a limited set of temporal parameters or trunk and arm swing velocities. This approach has been widely used in older adults or individuals with mobility impairments [28,29,30,31,32,33,34] but has been less extensively investigated in PD [2,5,35,36,37]. As noted in a recent systematic review [9], current single-sensor protocols primarily provide temporal, spatiotemporal or pelvic-related measures, rather than a comprehensive, multisegmental biomechanical characterization. Although several studies have investigated individual biomechanical domains, including trunk kinematics [2], axial rotation during turning [22], arm swing [38], pelvic motion [39], or upper-limb joint kinematics [40], these components have generally been analyzed in isolation rather than within an integrated whole-body framework. Consequently, detailed analyses of upper- and lower-body kinematics, joint- and plane-specific movement patterns, and frontal-plane control remain scarce [2,35,36,41,42]. This limitation is particularly relevant because frontal-plane biomechanics, despite their recognized role in mediolateral stability and turning performance, have received considerably less attention than sagittal-plane parameters [22,43]. In addition, only a limited number of studies [3,6,40] have compared individuals across different modified Hoehn and Yahr (mH&Y) stages, restricting our understanding of stage-associated biomechanical differences, particularly in the OFF-medication state. The present study addresses these methodological gaps by combining a phase-specific, multisegment biomechanical analysis of the iTUG with assessment in the OFF-medication state, and comparisons across early mH&Y stages and age-matched healthy controls. Specifically, this study aimed to identify candidate biomechanical markers characterizing differences among early-stage PD subgroups and healthy controls through a phase-specific analysis of the iTUG, and to explore their potential value for objective mobility assessment.

## 2. Materials and Methods

### 2.1. Participants and Ethical Considerations

This cross-sectional observational study investigated biomechanical parameters of the iTUG test in people with PD and healthy controls and was reported according to the STROBE guidelines for observational studies [44].

The study was approved by the Human Research Ethics Committee of Rey Juan Carlos University (ENM095/25260220251782025), and informed consent was obtained from all participants prior to enrollment in the study. In addition, the study was performed in accordance with the principles of the Declaration of Helsinki.

Participants were recruited from local community centers and PD associations. The inclusion criteria for the PD group were as follows: (a) diagnosis of idiopathic PD based on the United Kingdom PD Society Brain Bank criteria; (b) mH&Y stages 1 (unilateral disease), 1.5 (unilateral disease with axial involvement), and 2 (bilateral disease without balance impairment) [45]; (c) current treatment with antiparkinsonian medication; (d) ability to walk independently for at least 10 m; and (e) absence of cognitive impairment (Mini-Mental State Examination score (MMSE) > 24) [46]. The exclusion criteria included atypical parkinsonism, other neurological disorders, history of deep brain stimulation, lower limb prostheses, previous lower limb orthopedic surgery, and any relevant visual, vestibular, psychiatric, or behavioral disorders. The control group was matched to the PD group for age, weight, and height. No significant between-group differences were observed in the main anthropometric characteristics (Table 1); therefore, no additional covariate adjustment was performed. Participants were included if they had no history of musculoskeletal injuries or lower limb surgery within the past year, and no coexisting neuromuscular disorders.

### 2.2. Sample Size Justification

Given the clinical and exploratory nature of the study, participants were recruited through consecutive nonprobability sampling over the full duration of the project. This strategy allowed the inclusion of all eligible participants available during the recruitment period. A post hoc sensitivity analysis was then performed to determine the minimum detectable effect size with the final sample. For a cross-sectional comparison among three independent groups, the final sample size of 79 participants provided 80% power at α = 0.05 to detect a moderate-to-large effect size (f = 0.36) for a one-way independent groups design approximating the Kruskal–Wallis’s test. Therefore, the study was considered adequately powered to detect moderate-to-large between-group differences, although smaller effects may have remained undetected.

### 2.3. Experimental Setting and Protocol

The data collection process was conducted at the Laboratory of Movement Analysis, Biomechanics, Ergonomics, and Motor Control (LAMBECOM) located at Universidad Rey Juan Carlos. Prior to the experiment, the participants were subjected to a clinical evaluation. Participants with PD were evaluated by a trained therapist using Part III of the Movement Disorder Society-Unified Parkinson’s Disease Rating Scale (MDS-UPDRS-III), the Freezing of Gait Questionnaire (FoGQ), and the mH&Y [47,48,49]. All assessments were performed during the same session in the practically defined OFF-medication state, following an overnight withdrawal of the participants’ regular antiparkinsonian medication (minimum 12 h after the last dose of levodopa or other antiparkinsonian medication).

After clinical evaluation, participants performed the iTUG test. They were instructed to stand up from a chair, walk 3 m at their maximum safe speed, perform a 180° turn, return to the chair, and sit down. Prior to data collection, the procedure was explained and demonstrated by a trained healthcare professional. Participants were not explicitly instructed to swing their arms, allowing natural upper limb movement patterns to emerge.

Each participant completed four trials, a number selected to maximize the capture of representative movement events while minimizing fatigue, particularly in individuals with motor impairments. A trial-to-trial reliability analysis was performed to determine the consistency of the measurements and to verify whether data from the trials could be pooled. Reliability estimates were interpreted according to established guidelines [50].

### 2.4. Data Acquisition

The iTUG test was analyzed using a synchronized multimodal sensing framework combining video, optoelectronic motion capture, and inertial measurement systems.

The optoelectronic system consisted of eight infrared cameras (Vicon Vero 2.2, Vicon Motion Systems Ltd., Oxford, UK) operating at a sampling frequency of 100 Hz. Data were acquired using Vicon Nexus software (version 2.15), configured to record gait along an 11 m walkway. In addition, two synchronized video cameras (Blackfly S USB3, FLIR Systems Inc., Wilsonville, OR, USA) captured frontal and sagittal views of the task, enabling visual inspection and overlay of the reconstructed kinematic model onto the video recordings. Sixteen reflective markers were placed on the anatomical landmarks according to the Vicon Lower-Body Plug-in Gait model [51]. Initial contact and toe-off events were manually identified in Vicon Nexus, enabling the definition of gait cycles.

The inertial system consisted of seventeen MTw Awinda inertial measurement units (IMUs) (Xsens Technologies, Enschede, The Netherlands), which recorded triaxial accelerometer, gyroscope, and magnetometer data at 60 Hz from multiple body segments. Sensors were placed on the head, sternum, pelvis, and, bilaterally, the shoulders, upper arms, forearms, hands, thighs, shanks, and feet according to the manufacturer’s guidelines [52]. Temporal alignment between the optoelectronic and inertial data streams was achieved using a hardware trigger that simultaneously started recording on both the Vicon Nexus and Xsens MVN Analyze (version 2024) systems. Any residual misalignment between the two systems was subsequently corrected by aligning the vertical position of the foot (heel marker for the optoelectronic system and foot segment for the inertial system) on a per-trial basis.

Kinematic data (segment accelerations, velocities, and positions) were obtained using Xsens MVN Analyze, which estimates segment positions and orientations by integrating acceleration and angular velocity signals through its proprietary sensor-fusion algorithm. Optoelectronic trajectories were reconstructed in Vicon Nexus using the manufacturer’s default processing pipeline. No additional digital filtering was applied during post-processing. To reduce drift introduced by inertial position estimation and to harmonize coordinate frames between systems, Xsens data were reoriented using principal component analysis. For each trial, principal component analysis was applied to the horizontal trajectory data to determine the primary direction of progression, which was defined as the anteroposterior axis. Pelvis trajectories were used for the inertial system, whereas right heel marker trajectories were used for the optoelectronic system. Trial quality was assessed through expert inspection for residual drift and reconstruction artifacts [53].

### 2.5. TUG Segmentation and Outcome Measures

To obtain a comprehensive biomechanical representation of the iTUG, the test was segmented into six phases according to the previous literature [9,54]. All event labeling and phase identification were performed by a single experienced operator using synchronized video recordings and kinematic trajectories within Vicon Nexus software.

**Figure 1:**
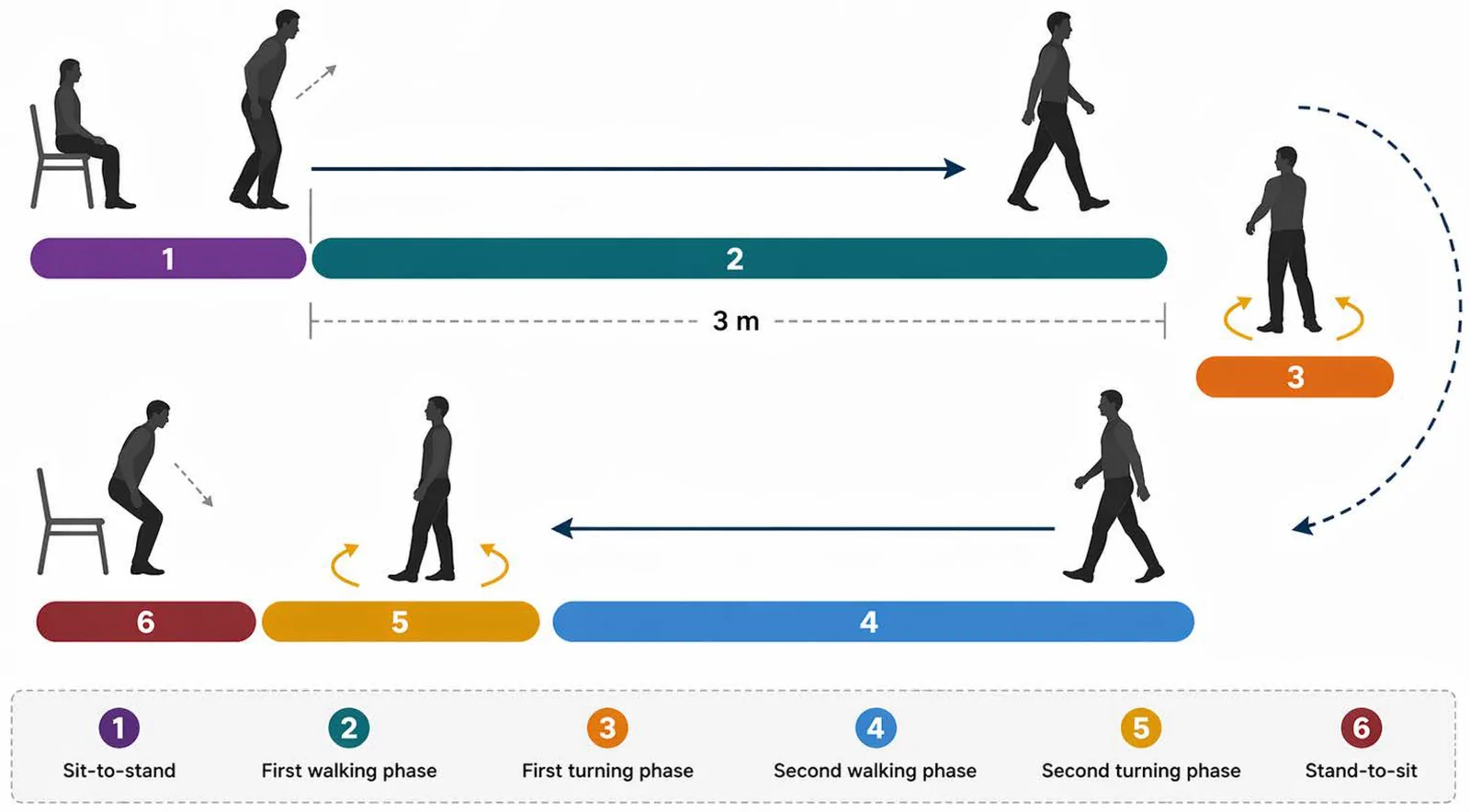
Six-phase segmentation of the iTUG test. Schematic representation of the six phases analyzed during the iTUG: (**1**) sit to stand, (**2**) first walking phase, (**3**) first turning phase, (**4**) second walking phase, (**5**) second turning phase, and (**6**) stand to sit. Blue arrows indicate the direction of walking, whereas orange arrows indicate the 180° turning movements.

The iTUG was segmented into six phases: (1) sit-to-stand, defined as the interval between the onset of forward trunk flexion in the seated position and the first heel strike after achieving an upright posture; (2) first walking phase, defined as the interval between the first heel strike after achieving an upright posture and the last frame before the foot trajectory deviated from the straight walking path toward the turning direction; (3) first turning phase, defined as the interval between the last frame before the foot trajectory deviated from the straight walking path toward the turning direction and the first frame after completion of the directional change, when the foot resumed straight forward progression along the return walking path; (4) second walking phase, defined as the interval between the end of the first turning phase and the last frame before the foot trajectory deviated toward the second turn; (5) second turning phase, defined as the interval between the last frame before the foot trajectory deviated from the straight walking path and the first frame after completion of the directional change, when both feet were aligned with the chair and before the onset of knee flexion for sitting; and (6) stand-to-sit, defined as the interval from the onset of knee flexion to a stable seated posture. A schematic representation of the phase segmentation is provided in Figure 1.

Outcome measures were computed for the entire iTUG as well as for each individual phase. These included: (1) TUG times: total iTUG duration and phase-specific durations (sit to stand, first walking phase, first turning phase, second walking phase, second turning phase, and stand to sit); (2) spatiotemporal parameters: step width and turning area, as well as single- and double-support time (expressed as percentages of the gait cycle), stride length, stride time, and walking speed, calculated separately for the more affected and less affected sides in participants with PD and compared with controls; (3) gait variability: coefficients of variation (CV) [12] of step width, stride length, stride time, and walking speed; and (4) kinematic parameters: upper- and lower-body joint kinematics, including shoulder and elbow asymmetry indices, and range of motion (ROM) of the trunk, shoulder, elbow, hip, knee, and ankle.

Upper-body analysis focused on trunk ROM in the sagittal and frontal planes, whereas shoulder and elbow kinematics were examined in the sagittal plane, along with their corresponding asymmetry indices, in accordance with the previous literature [33]. Lower-body analysis included hip ROM in both sagittal and frontal planes, while knee and ankle kinematics were assessed in the sagittal plane, based on evidence indicating higher reliability and lower measurement error in these planes for clinical interpretation [55].

In people with PD, all unilateral kinematic variables were analyzed separately for the more affected and less affected sides, defined through clinical examination and patient-reported symptom predominance. In healthy controls, preliminary analyses showed no relevant differences between dominant and non-dominant sides; therefore, the dominant side was used for comparison.

### 2.6. Data Processing, Outcome Computation, Quality Control, and Trial-to-Trial Reliability Analysis

Metrics were computed using Python 3.11 (Python Software Foundation, Wilmington, DE, USA) following a standardized data processing pipeline and converted into the Eurobench standardized data format [56]. Kinematic variables (segment orientations, joint angles, and positions) were extracted using the proprietary processing engines of Vicon Nexus 2.15 and Xsens and Xsens MVN Analyze. The exported kinematic and spatiotemporal data were subsequently processed using a custom Python pipeline for temporal synchronization, phase segmentation, coordinate-frame reorientation, gait-cycle quality screening and normalization, and computation of the outcome measures. The pipeline was implemented using standard open-source libraries (NumPy, pandas, SciPy, PyYAML, and openpyxl).

All trial-level biomechanical outcomes underwent a quality-control procedure to identify and remove measurements affected by reconstruction errors, inertial-recording artifacts, or non-physiological values. Exclusions were performed on an outcome-specific basis; therefore, a given trial could be retained for some variables while being excluded from others. Variables requiring gait-cycle aggregation, particularly coefficients of variation, were calculated only when enough valid gait cycles remained after quality screening.

From these processed data, spatiotemporal, kinematic, variability, and asymmetry metrics were derived. Step width was calculated as the mean mediolateral distance between the feet during double support, whereas stride length was computed as the anteroposterior distance between successive heel strikes of the same foot. Single- and double-support times were derived and expressed as percentages of the gait cycle. Walking speed was calculated over the walking segments of the iTUG by dividing the total walking distance (6 m, corresponding to the 3 m outward and return paths) by the time required to complete these phases. Turning area was quantified as the area enclosed by the projection of the center-of-mass trajectory onto the ground during turning. Joint range of motion (ROM) was calculated as the mean difference between the maximum and minimum joint angles across valid gait cycles [57].

Kinematic variables were calculated exclusively during the walking phases of the iTUG. Gait variability was quantified using coefficients of variation (CV), calculated as (SD/mean) × 100 across valid cycles for each participant. Asymmetry indices (AI) were computed as 100 × |LAS − MAS|/max (LAS, MAS), where LAS and MAS represent the less affected and more affected sides in PD, respectively. In healthy controls, the left and right sides were used equivalently [58].

For the primary analyses, participant-level outcomes were calculated as the mean of all valid trial-level measurements available for the corresponding variable. Consequently, participants contributed to the between-group analyses whenever at least one valid trial-level measurement was available for the outcome of interest. Because the number of valid observations differed across outcomes after quality control, outcome-specific sample sizes are reported in the corresponding tables.

To determine whether measurements could be pooled across trials, trial-to-trial reliability was assessed separately for each biomechanical outcome. Reliability analyses were performed using a one-way random-effects model fitted by restricted maximum likelihood. Participants with at least two valid trials were included in these analyses, as repeated observations are required to estimate within-participant variability. Reliability was expressed as ICC(1, 1) for a single trial, and ICC(1, *k*) for the participant-level mean. Because trial availability varied among participants, *k* represented the effective number of trials (*k*_eff_), calculated as the harmonic mean of the participant-specific numbers of valid trials. Corresponding 95% confidence intervals were estimated using participant-level bootstrap resampling.

Complete outcome-specific reliability estimates, sample sizes, trial availability, and 95% confidence intervals are reported in Supplementary Materials Table S1.

### 2.7. Statistical Analysis

All statistical analyses were performed using SPSS software (IBM SPSS Statistics, version 29.0, IBM Corp., Armonk, NY, USA) [59]. Normality was assessed using the Shapiro–Wilk test. As several variables violated parametric assumptions and given the relatively small sample size, nonparametric analyses were employed.

Between-group differences among controls, participants with PD at mH&Y stages 1–1.5, and those in stage 2 were examined using the Kruskal–Wallis test. When significant omnibus group differences were identified, post hoc pairwise comparisons were conducted using the Mann–Whitney U test.

To control Type I error across the three pairwise comparisons conducted for each outcome, Bonferroni correction was applied, resulting in an adjusted significance threshold of *p* < 0.0167. The reported Mann–Whitney U test *p*-values were unadjusted two-sided values assessed against this threshold, whereas the significance threshold for the omnibus Kruskal–Wallis tests was *p* < 0.05. No multiplicity correction was applied across the omnibus tests conducted for the different outcomes. Therefore, given the number of variables examined, these analyses should be considered exploratory and hypothesis-generating.

Analyses were performed using the available valid observations for each outcome, without imputation of unavailable values. Because unavailable measurements resulted from technical recording limitations and predefined quality-control exclusions rather than participant attrition, an available case approach was adopted. Consequently, the number of observations could vary across outcomes and is reported in the corresponding tables.

The global effect size for the Kruskal–Wallis test was estimated using epsilon squared (ε^2^ = [*H* − *k* + 1]/ [*N* − *k*], where *H* is the Kruskal–Wallis statistic, *k* is the number of groups, and *N* is the outcome-specific total number of valid observations). Values of ε^2^ ≈ 0.01, ≈0.08, and ≥0.14 were interpreted as small, moderate, and large effects, respectively. For post hoc pairwise comparisons, effect sizes were calculated as *r* = ∣*Z*∣/√*N*, where *N* represents the combined number of valid observations in the two groups being compared. Values of *r* ≈ 0.10, ≈0.30, and ≥0.50 were interpreted as small, moderate, and large effects, respectively [60].

## 3. Results

### 3.1. Participant Characteristics

A total of 79 participants were included in the study (38 with PD and 41 healthy controls). All participants completed the four-trial iTUG protocol, yielding 316 recorded trials. No participant withdrew, and no complete trial was excluded. Nevertheless, outcome-specific trial-level measurements were occasionally unavailable after quality control because of reconstruction errors, inertial-recording artifacts, or an insufficient number of valid gait cycles required for the calculation of selected variables. Consequently, outcome-specific sample sizes varied according to the biomechanical measure analyzed.

Participant characteristics are presented in Table 1. Healthy controls were well matched to the PD cohort for age, sex, height, and weight, with no significant between-group differences. Within the PD group, 15 participants were classified as mH&Y stages 1–1.5 and 23 as stage 2. Participants with stage 2 PD showed numerically greater disease duration and higher MDS-UPDRS III and FoGQ scores; however, these differences were not statistically significant. Although the proportion of males was descriptively higher in the mH&Y 2 subgroup, hand dominance and fall history were comparable across groups.

### 3.2. Trial-to-Trial Reliability

Trial-to-trial reliability was moderate to excellent across biomechanical outcomes, with ICC(1, 1) values ranging from 0.56 to 0.94 (Supplementary Materials Table S1). Reliability improved when participant-level means were calculated from all available valid trials, yielding good-to-excellent ICC(1, *k*) values for all outcomes (0.81–0.98). Sit-to-stand time showed the lowest reliability [ICC(1, 1) = 0.56, 95% CI: 0.27–0.66; ICC(1, *k*) = 0.81, 95% CI: 0.57–0.87]. Outcome-specific sample sizes and trial availability varied across variables. Complete reliability results, including 95% confidence intervals, are reported in Supplementary Materials Table S1.

### 3.3. iTUG Temporal Parameters

Total iTUG duration showed a significant group effect (*H* = 13.81, *p* = 0.001) with a large effect size. Controls performed the test faster than the mH&Y 2 PD group [7.83 (1.77) s vs. 8.92 (2.14) s], remaining significant after Bonferroni correction, with a moderate effect size. No significant differences were observed between controls and the mH&Y 1–1.5 PD group or between PD subgroups.

Phase-specific analysis revealed that sit-to-stand duration did not differ across groups, whereas differences emerged in walking and turning phases (Table 2). During the first walking phase, controls were faster than the mH&Y 2 PD group [2.69 (0.48) s vs. 2.90 (0.80) s], with a moderate effect size. The first turning phase showed marked differences, with longer durations in the mH&Y 2 PD group compared with controls [1.88 (0.69) s vs. 1.54 (0.31) s], with a moderate-to-large effect size. Notably, this was the only phase that also differentiated controls from the mH&Y 1–1.5 PD group, with a moderate effect size. Similar patterns were observed in the second walking phase (1.34 s vs. 1.53 s), second turning phase (1.41 s vs. 1.65 s), and stand-to-sit phase (0.96 s vs. 1.30 s), all showing moderate effect sizes, with all differences remaining significant after Bonferroni correction between controls and the mH&Y 2 PD group. Participants with PD in the mH&Y 1–1.5 group consistently showed intermediate values, although none of these comparisons remained significant after Bonferroni adjustment.

### 3.4. Spatiotemporal Parameters and Coefficients of Variation

Spatiotemporal parameters calculated during the walking phases are summarized in Table 3. Step width did not differ between groups. The turning area showed a significant group effect, with a small effect size, although no pairwise differences remained significant after Bonferroni correction.

On the more affected side, single-support percentage was reduced in the mH&Y 2 PD group compared with controls, with a moderate effect size. Walking speed was also lower in the mH&Y 2 PD group than in controls (1.13 m/s vs. 1.40 m/s), showing a moderate effect size.

On the less affected side, double-support percentage was significantly higher in the mH&Y 2 PD group compared with controls [23.21% (11.15) vs. 15.42% (6.79)], with a moderate effect size. Similarly, stride length was reduced [0.98 (0.26) m vs. 1.16 (0.16) m], and walking speed was lower [1.10 (0.46) m/s vs. 1.40 (0.22) m/s] in the mH&Y 2 PD group compared with controls, both showing moderate effect sizes.

Gait variability, expressed as coefficients of variation (CV), did not differ significantly between groups for any parameter (Table 4).

### 3.5. Upper Body Kinematics

**Figure 2:**
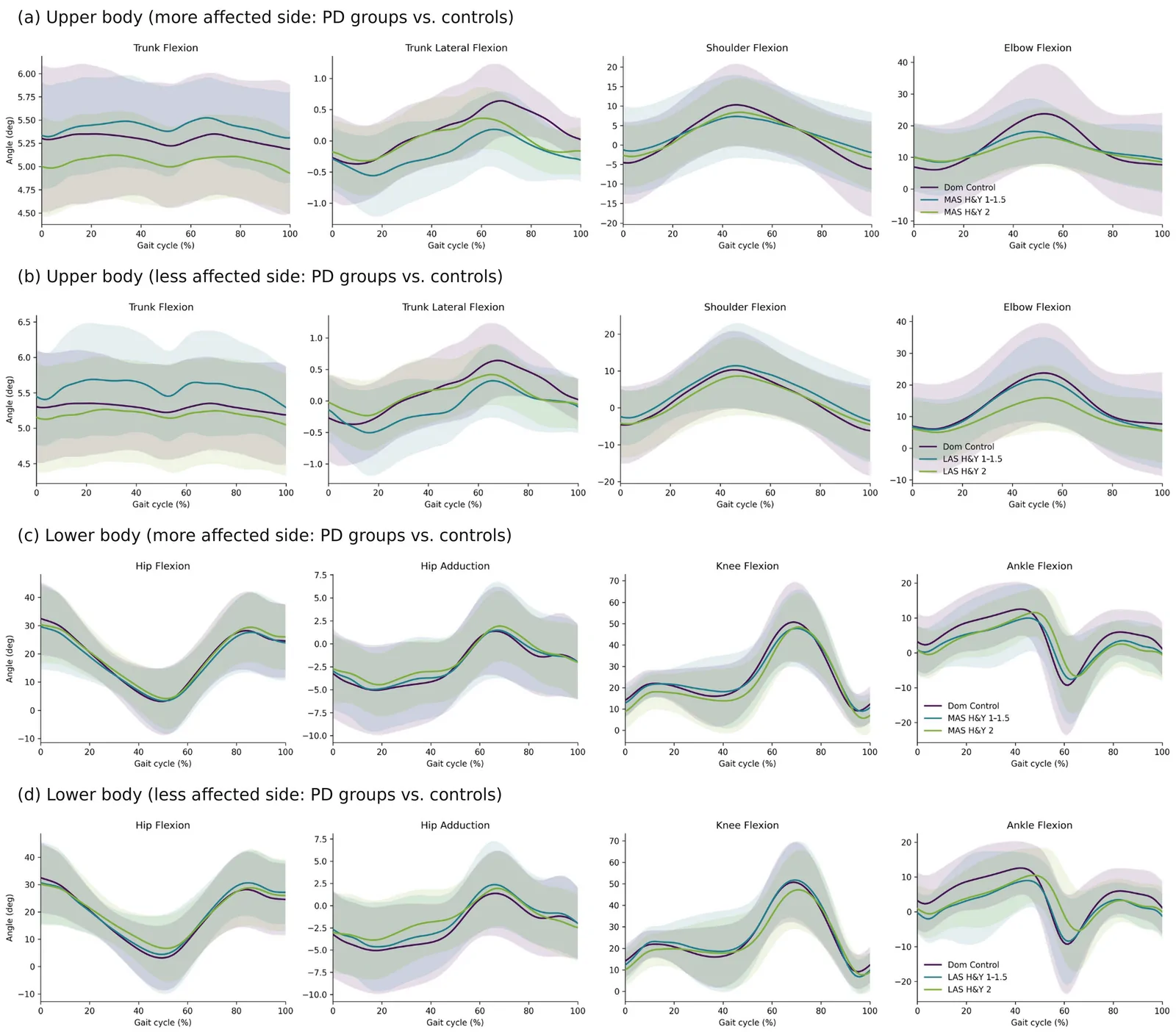
Kinematics plots across the iTUG walking phases. Kinematic profiles are presented for participants with PD in mH&Y stages 1–1.5 and 2, and controls. Panels show: (**a**) upper body kinematics on the more affected side, (**b**) upper body kinematics on the less affected side, (**c**) lower body kinematics on the more affected side, and (**d**) lower body kinematics on the less affected side. Curves represent the mean kinematic profiles obtained by averaging all gait cycles within each group after time-normalization to 100% of the gait cycle. Solid lines represent mean values, and shaded areas indicate standard deviation. Purple, blue, and green represent controls, participants with PD at mH&Y stages 1–1.5, and participants with PD at mH&Y stage 2, respectively.

Upper-body kinematic results are presented in Table 5 and Figure 2. No significant differences were observed in shoulder asymmetry. However, elbow asymmetry was significantly greater in the mH&Y 1–1.5 PD group compared with controls [38.14% (29.86) vs. 26.85% (18.30)], with a moderate effect size.

On the more affected side, trunk ROM was reduced in the mH&Y 2 PD group in both sagittal [0.42° (0.26) vs. 0.65° (0.31)] and frontal planes [1.20° (0.27) vs. 1.50° (0.36)], showing moderate effect sizes. Elbow ROM was also markedly reduced in the mH&Y 2 PD group compared with controls [14.83° (14.27) vs. 30.73° (18.21)], with a large effect size. Shoulder ROM showed a significant group effect, although no between-group differences remained significant after correction.

On the less affected side, sagittal trunk ROM was reduced in the mH&Y 2 PD group compared with both controls and the mH&Y 1–1.5 PD group, with moderate-to-large effect sizes. Notably, this was the only upper-body variable that significantly differed between the two PD groups after Bonferroni correction. Frontal trunk ROM was also reduced in the mH&Y 2 PD group compared with controls, showing a moderate effect size. Shoulder and elbow ROM on the less affected side did not differ between groups.

### 3.6. Lower Body Kinematics

Lower-body kinematic results are presented in Table 6 and Figure 2. On the more affected side, sagittal hip and knee ROM did not differ between groups. However, frontal hip ROM was reduced in the mH&Y 2 PD group compared with controls [9.25° (2.13) vs. 11.60° (3.28)], with a moderate effect size. Ankle ROM was also reduced in the mH&Y 2 PD group [26.79° (11.77) vs. 33.70° (16.01)], showing a moderate effect size.

On the less affected side, no significant differences were observed for hip or knee ROM. Ankle ROM showed a significant overall group effect (*p* = 0.026), with a small effect size, although post hoc comparisons did not remain significant after correction.

Overall, direct comparisons between participants with PD at mH&Y stages 1–1.5 and 2 revealed relatively few significant differences after Bonferroni correction. Among all biomechanical variables analyzed, trunk sagittal ROM on the less affected side was the only parameter that showed a significant difference between the two PD subgroups, whereas most other significant findings primarily distinguished participants with mH&Y stage 2 from healthy controls.

## 4. Discussion

**Figure 3:**
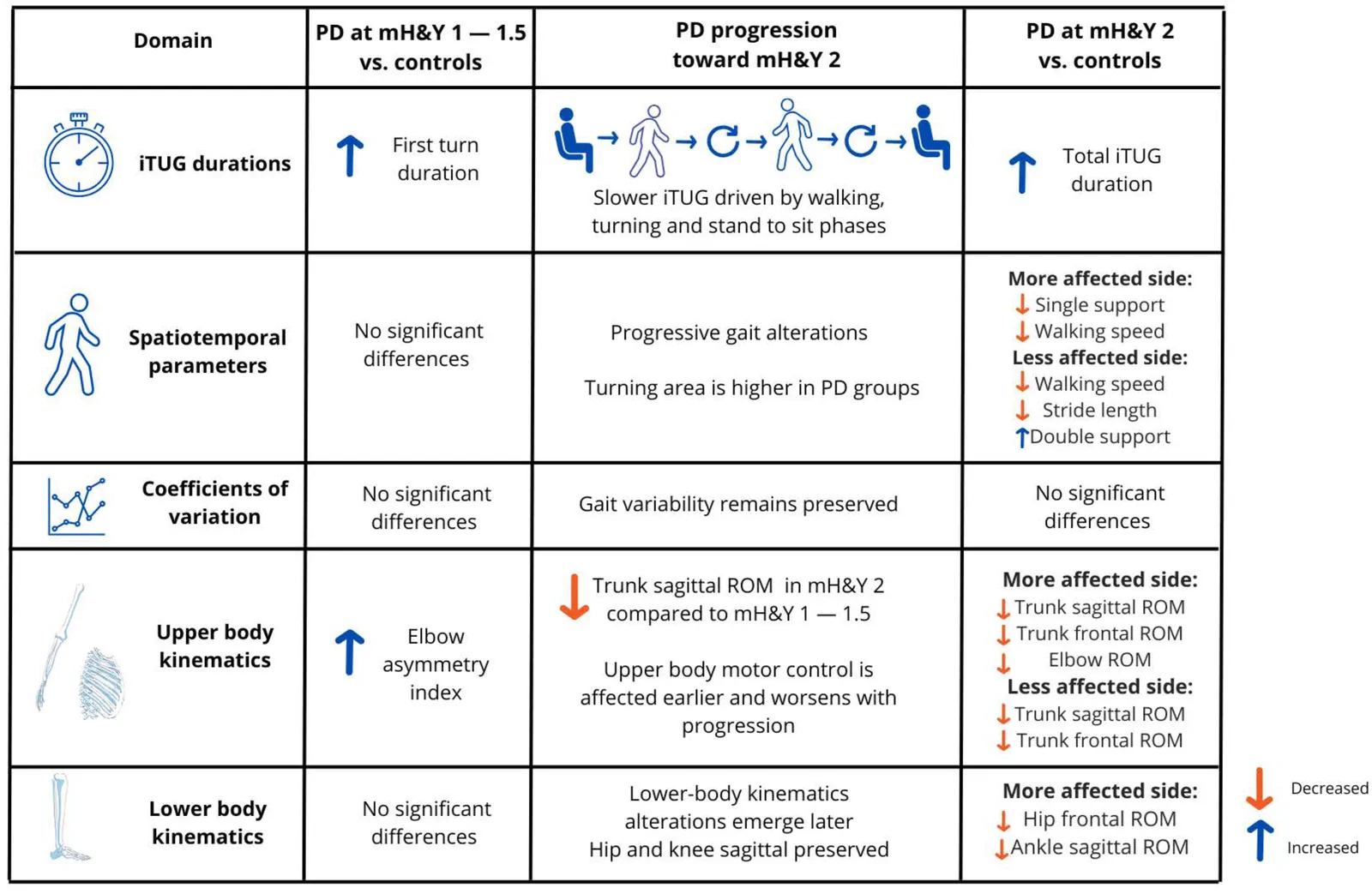
Summary of the main candidate iTUG alterations across early PD stages. The figure presents the temporal, spatiotemporal, gait-variability, and kinematic group differences observed during the iTUG. Candidate early-stage alterations were mainly related to turning performance and upper-body control, whereas lower-body and spatiotemporal gait differences were more evident at mH&Y stage 2. Blue upward arrows indicate increased values, orange downward arrows indicate reduced values, and “No significant differences” indicates the absence of statistically significant between-group differences.

The present study provides a comprehensive biomechanical characterization of motor performance during the iTUG in people with early stages of PD evaluated in the OFF-medication state. The main findings are summarized in Figure 3.

Consistent with the previous literature, total iTUG duration showed limited sensitivity to early disease-related changes [3,35], despite retaining clinical value for fall-risk stratification [6]. In this context, segmenting the iTUG into six phases could enhance its sensitivity, as supported by previous methodological studies [1,9,61,62]. These findings reinforce the limitations of relying on global outcomes and suggest that instrumented approaches capable of capturing phase-specific and multisegmental biomechanical features may provide additional biomechanical information beyond conventional TUG metrics [20,36,63,64].

Phase-specific analysis showed that walking and turning phases were primarily affected, whereas sit-to-stand transitions remained relatively preserved. Although people with PD required more time to complete the iTUG than controls, the most pronounced differences were found in the walking and turning phases, while sit-to-stand transitions remained comparatively preserved. This is consistent with previous findings in PD [65,66], whereas in other populations, such as people with dementia, sit-to-stand performance is often more severely impaired [64]. This suggests that basic transitional motor capacity remains relatively preserved in the early stages of PD, whereas locomotor tasks and changes in direction reveal more pronounced deficits [65,67]. Among all phases, turning emerged as the most sensitive marker of early motor impairment. Notably, it was the only phase that differentiated controls from participants with PD at mH&Y stages 1–1.5. From a biomechanical perspective, turning requires coordinated axial rotation, anticipatory postural adjustments, and fine control of whole-body angular momentum; all functions affected in people with PD [3,68,69]. The prolonged turning durations, together with the increased turning area observed at the group level, suggest reduced axial mobility and impaired mediolateral balance control, as also reported in previous studies [17,22]. Notably, these alterations were evident even when straight-line walking remained relatively stable, underscoring the value of examining directional change behavior for detecting emerging motor impairments, in line with previous findings [34,62]. This finding has potential clinical implications, as turning performance may help identify early movement control deficits not captured by linear gait assessments and may represent a relevant target for physiotherapy interventions [15].

Spatiotemporal findings further support a more pronounced stability-oriented gait strategy in participants at the mH&Y stage, characterized by reduced walking speed and stride length, together with increased double support and reduced single support. These adaptations may present compensatory mechanisms aimed at enhancing stability and have previously been associated with impaired balance control and increased fall risk [25,43,70]. Their inconsistent presence at mH&Y stages 1–1.5 suggests that spatiotemporal parameters may be less sensitive to subtle deficits at the earliest stages.

In contrast to the identified spatiotemporal changes, gait variability did not differ significantly between groups. This finding suggests that step-to-step regularity may be maintained in early PD, at least during short and structured tasks such as the iTUG. Alterations in movement amplitude and intersegmental coordination may therefore be detectable even in the absence of increased gait variability [71].

Upper body kinematics revealed a distinct proximal-distal and stage-dependent pattern of motor involvement. In our study, elbow asymmetry increased even in participants with PD at mH&Y stages 1–1.5, whereas shoulder asymmetry remained unchanged, suggesting that distal upper limb control may be more vulnerable early in the disease course [72]. Increased arm swing asymmetry has also been described as an early sign of parkinsonian gait disturbance, often preceding alterations in lower limb locomotion [14]. This is consistent with previous studies reporting reduced arm swing amplitude and early asymmetry in upper limb movement [38,73], although some authors suggest that proximal and distal segments may deteriorate more simultaneously [40]. Previous studies suggest that, as the disease progresses, early distal alterations appear to be accompanied by more pronounced axial involvement. In our study, people with PD at mH&Y stage 2 showed markedly reduced trunk ROM in both sagittal and frontal planes, consistent with greater axial rigidity [5,15,22]. Importantly, trunk sagittal ROM was the only upper body parameter that differentiated between people with PD across early mH&Y stages, suggesting that axial sagittal control may be particularly sensitive to early disease progression. Concomitantly, elbow ROM was also substantially reduced in this subgroup, suggesting a diminished contribution of the upper limbs during gait, consistent with previous studies [14,54]. Notably, on the less affected side, trunk kinematic alterations were already evident, whereas shoulder and elbow motion remained relatively preserved. This asymmetric pattern, characterized by bilateral axial limitation combined with distal asymmetry, supports the notion that alterations in trunk mobility and elbow kinematics may represent early biomechanical markers of functional mobility impairment during the iTUG, in agreement with previous findings [14,72].

Lower body kinematic results revealed a joint-specific pattern of impairment predominantly observed at mH&Y stage 2 [17,44]. On the more affected side, individuals with PD at mH&Y stage 2 exhibited reduced frontal-plane hip ROM and sagittal-plane ankle ROM compared with controls, whereas sagittal hip and knee ROM remained relatively preserved. Consistent with the previous literature, sagittal knee kinematics appeared unaffected in the early stages of the disease [74]. In contrast, alterations at the ankle level emerged as a more prominent finding, with reduced sagittal-plane ROM observed in our cohort, differing from some earlier reports [74]. A consistent feature in the mH&Y 2 group was the impairment of frontal-plane control. Reduced trunk and hip motion in the frontal plane, together with increased double support and longer turning durations, suggest compromised mediolateral stability and impaired balance regulation [43,75]. Given the central role of frontal-plane joint motion in maintaining mediolateral stability, reduced hip ROM in this plane may contribute to the altered turning strategies and greater reliance on double-support phases observed in our participants. Previous authors have proposed that these adaptations are likely to represent compensatory mechanisms aimed at improving stability during walking and turning [25,70]. Regarding ankle motion, the reduction in sagittal-plane ROM may reflect greater distal motor involvement at mH&Y stage 2. Furthermore, the OFF-medication condition assessed in the present study may have contributed to unmasking subtle distal motor deficits that are not consistently identified under medicated conditions.

This study has several limitations. First, all assessments were intentionally performed in the OFF-medication state to characterize the unmasked motor phenotype and maximize sensitivity to subtle motor impairments. While this approach reduced medication-related variability, the absence of complementary ON-medication assessments prevents determination of whether the observed biomechanical alterations reflect stable disease-related deficits or are partially responsive to dopaminergic treatment. Therefore, the clinical generalizability of the identified biomechanical markers should be interpreted cautiously. Future studies including both medication states may help identify which phase-specific alterations are treatment-sensitive and which remain stable across medication conditions.

From a clinical perspective, the findings observed in our study suggest the potential value of targeted assessment strategies. In participants with PD at mH&Y 1–1.5, conventional iTUG metrics may fail to detect subtle abnormalities. Consequently, more challenging or sensitive tasks, such as dual-task conditions, complex turning tasks, or continuous walking assessments, may be necessary to detect subtle deficits in this stage. These approaches may increase cognitive-motor demands and reduce compensatory capacity, thereby enhancing sensitivity to early-stage impairments [71,76,77]. Although the multimodal assessment protocol used in this study was designed for comprehensive biomechanical characterization rather than routine clinical implementation, the identified candidate biomechanical markers may support the future development of simplified assessment protocols based on fewer wearable sensors.

Second, participants were recruited through consecutive non-probability sampling, a common approach in clinical biomechanical research, but one that may limit sample representativeness and introduce selection bias. In addition, the laboratory-based assessment required specialized equipment, trained personnel, and offline data processing, limiting the immediate applicability of the protocol in routine clinical settings.

Third, the cross-sectional design precludes conclusions regarding disease progression. Furthermore, although the study was adequately powered to detect moderate-to-large effects, the sample size may have limited the ability to identify subtle biomechanical differences between participants at mH&Y stages 1–1.5 and 2. Consequently, the absence of significant differences for some outcomes should not be interpreted as evidence of equivalence between stages. This issue is particularly relevant given the limited literature examining biomechanical differences across the earliest stages of PD, where available studies have often relied on broader severity categories or mixed-stage cohorts [3,78,79,80,81]. Larger longitudinal studies are warranted to better characterize stage-related biomechanical changes during early disease progression.

Fourth, data availability varied across outcomes because some measurements did not satisfy predefined quality-control criteria or required enough valid gait cycles for computation. As a result, effective sample sizes were lower for selected outcomes, particularly gait-variability measures, which may have reduced statistical precision. Nevertheless, these procedures ensured that analyses were based exclusively on valid and physiologically plausible measurements.

Finally, multiple omnibus tests were conducted across several biomechanical domains without applying a study-wide multiplicity adjustment. Although this approach was adopted to preserve sensitivity in this exploratory investigation, it may have increased the risk of false-positive findings. Accordingly, the results should be considered hypothesis-generating and require confirmation in independent cohorts using prespecified primary outcomes and appropriate multiplicity-control procedures.

## 5. Conclusions

A phase-specific, multisegmental biomechanical analysis of the iTUG provides objective insights into functional mobility impairments in Parkinson’s disease beyond total task duration alone. Turning performance, frontal-plane control, and selected trunk, elbow, and ankle kinematic measures showed potentially relevant group differences and emerged as candidate biomechanical markers requiring further investigation, whereas stride-to-stride variability did not differ significantly between groups, suggesting that phase-specific biomechanical alterations may be more informative than gait variability in individuals with early-stage PD. Participants at mH&Y stages 1–1.5 exhibited only subtle alterations, suggesting that more demanding or sensitive tasks may be necessary to detect early deficits. These findings suggest that phase-specific, sensor-based mobility assessment may be useful for characterizing biomechanical differences between individuals with early PD and healthy controls, as well as stage-associated differences, in a cross-sectional setting. However, given the exploratory nature of the analyses and the number of outcomes examined, these findings require confirmation in future studies with prespecified primary outcomes and appropriate multiplicity control.

## Figures and Tables

**Table 1:** Demographic and clinical characteristics of the participants.

Variable	PD mH&Y 1–1.5 (*n* = 15)	PD mH&Y 2 (*n* = 23)	Controls (*n* = 41)	H/U	Sig.
Age (years)	64 (12); 64.6 ± 8.11	66 (16); 67.52 ± 8.46	67 (11); 68.02 ± 7.16	1.931	0.381
Height (cm)	162.0 (13.5); 162.6 ± 11.94	162.0 (10.0); 162.8 ± 7.64	165.0 (12.5); 164.2 ± 7.79	0.538	0.764
Weight (kg)	69.3 (16.2); 74.5 ± 21.9	73.0 (16.4); 70.8 ± 11.5	70.3 (14.0); 71.6 ± 13.6	0.082	0.960
Sex	7 M (46.7%); 8 F (53.3%)	17 M (73.9%); 6 F (26.1%)	23 M (56.1%); 18 F (43.9%)	—	—
Dominance	15 right (100%)	23 right (100%)	37 right (92.7%); 3 left (7.3%)	—	—
Falls (last year)	No: 13 (86.7%); Yes: 2 (13.3%)	No: 20 (87.0%); Yes: 3 (13.0%)	No: 38 (92.7%); Yes: 3 (7.3%)	—	—
Disease duration (months)	34 (40); 39.9 ± 28.3	52 (61); 70.8 ± 59.6	—	109.5	0.086
MDS-UPDRS-III	7 (7); 8.93 ± 4.48	11.5 (7); 11.09 ± 4.71	—	124	0.146
FoGQ	2 (4); 2.73 ± 2.43	4.5 (7); 4.5 ± 3.52	—	119.5	0.111

Values are presented as median (interquartile range) and mean ± standard deviation, unless otherwise indicated. H represents the Kruskal–Wallis test for comparisons among the three groups, whereas U represents the Mann–Whitney U test for comparisons between PD subgroups. PD: Parkinson’s disease; mH&Y: modified Hoehn and Yahr stage; UPDRS-III: Movement Disorder Society–Unified Parkinson’s Disease Rating Scale, Part III; FoGQ: Freezing of Gait Questionnaire; M: male; F: female. Statistical significance was set at (*p* < 0.05).

**Table 2:** Comparison of iTUG temporal parameters among groups.

Parameter	Group	Median	IQR	*H*	ε^2^	Sig.	Comparison	*U*	*r*	Sig.
Total iTUG (s)	Control (*n* = 41)	7.831	1.77	13.814	0.16	0.001 *	Control vs. PD 1–1.5	192	0.29	0.033
	PD 1–1.5 (*n* = 15)	9.216	1.79				Control vs. PD 2	217	0.44	<0.001 **
	PD 2 (*n* = 23)	8.915	2.14				PD 1–1.5 vs. PD2	148	0.12	0.464
Sit to stand (s)	Control (*n* = 41)	1.182	0.17	1.781	0.01	0.410				
	PD 1–1.5 (*n* = 15)	1.266	0.34							
	PD 2 (*n* = 23)	1.227	0.34							
First Walking (Go) (s)	Control (*n* = 41)	2.694	0.48	7.196	0.07	0.027 *	Control vs. PD 1–1.5	222	0.21	0.114
	PD 1–1.5 (*n* = 15)	2.873	0.71				Control vs. PD 2	292	0.31	0.012 **
	PD 2 (*n* = 23)	2.900	0.80				PD 1–1.5 vs. PD2	146	0.13	0.429
First turn (s)	Control (*n* = 41)	1.536	0.31	13.159	0.15	0.001 *	Control vs. PD 1–1.5	177.5	0.32	0.015 **
	PD 1–1.5 (*n* = 15)	1.828	0.63				Control vs. PD 2	237	0.41	0.001 **
	PD 2 (*n* = 23)	1.875	0.69				PD 1–1.5 vs. PD2	144.5	0.14	0.403
Second Walking (Return) (s)	Control (*n* = 41)	1.341	0.45	8.114	0.08	0.017 *	Control vs. PD 1–1.5	210	0.24	0.071
	PD 1–1.5 (*n* = 15)	1.520	0.43				Control vs. PD 2	283.5	0.33	0.009 **
	PD 2 (*n* = 23)	1.530	0.68				PD 1–1.5 vs. PD2	152	0.10	0.540
Second turn (s)	Control (*n* = 41)	1.413	0.30	9.985	0.11	0.007 *	Control vs. PD 1–1.5	195	0.28	0.037
	PD 1–1.5 (*n* = 15)	1.650	0.57				Control vs. PD 2	265	0.36	0.004 **
	PD 2 (*n* = 23)	1.650	0.59				PD 1–1.5 vs. PD2	156	0.08	0.622
Stand to sit (s)	Control (*n* = 41)	.957	0.29	10.665	0.11	0.005 *	Control vs. PD 1–1.5	224	0.21	0.122
	PD 1–1.5 (*n* = 15)	1.150	0.49				Control vs. PD 2	247	0.39	0.002 **
	PD 2 (*n* = 23)	1.295	0.53				PD 1–1.5 vs. PD2	125	0.23	0.156

Values are presented as median and interquartile range (IQR). * Statistical significance for the omnibus comparisons was determined using the Kruskal–Wallis test (*p* < 0.05), and the global effect size was estimated using epsilon squared (ε2). ** Post hoc pairwise comparisons were conducted using the Mann–Whitney U test. Reported *p*-values are unadjusted two-sided values and were assessed against the Bonferroni-corrected significance threshold (α = 0.0167). Effect sizes were calculated using *r* = (*Z*/√*N*), where *N* represents the combined number of observations in the two groups being compared. Bold values indicate statistically significant results. Sample sizes were consistent across all outcomes: controls, *n* = 41; PD mH&Y 1–1.5, *n* = 15; and PD mH&Y 2, *n* = 23.

**Table 3:** Spatiotemporal gait parameters during the iTUG walking phases.

Parameter	Group	Median	IQR	*H*	ε^2^	Sig.	Comparison	*U*	*r*	Sig.
Step width (m)	Control (*n* = 36)	0.1195	0.05	3.139	0.02	0.208				
	PD 1–1.5 (*n* = 14)	0.1357	0.06							
	PD 2 (*n* = 23)	0.1501	0.06							
Turning area (m2)	Control (*n* = 41)	0.0538	0.06	7.175	0.07	0.028 *	Control vs. PD 1–1.5	201	0.26	0.049
	PD 1–1.5 (*n* = 15)	0.1506	0.27				Control vs. PD 2	305	0.29	0.020
	PD 2 (*n* = 23)	0.1236	0.18				PD 1–1.5 vs. PD2	171	0.01	0.964
**More affected side/controls**										
Double Support (%)	Control (*n* = 33)	15.424	6.788	4.070	0.03	0.131				
	PD 1–1.5 (*n* = 12)	20.999	13.628							
	PD 2 (*n* = 20)	22.374	10.978							
Single support (%)	Control (*n* = 32)	38.651	4.133	7.719	0.10	0.021 *	Control vs. PD 1–1.5	169	0.09	0.544
	PD 1–1.5 (*n* = 12)	40.297	8.276				Control vs. PD 2	139	0.40	0.005 **
	PD 2 (*n* = 17)	36.701	5.964				PD 1–1.5 vs. PD2	67	0.29	0.121
Stride length (m)	Control (*n* = 33)	1.1625	0.1570	5.774	0.06	0.056				
	PD 1–1.5 (*n* = 12)	1.0918	0.3083							
	PD 2 (*n* = 20)	0.9814	0.2533							
Stride time (s)	Control (*n* = 33)	0.8666	0.1678	3.881	0.03	0.144				
	PD 1–1.5 (*n* = 12)	0.9861	0.0680							
	PD 2 (*n* = 20)	0.9218	0.2026							
Walking speed (m/s)	Control (*n* = 33)	1.4038	0.2160	8.971	0.11	0.011 *	Control vs. PD 1–1.5	111	0.33	0.026
	PD 1–1.5 (*n* = 12)	1.1266	0.3181				Control vs. PD 2	189	0.36	0.010 **
	PD 2 (*n* = 20)	1.1276	0.4738				PD 1–1.5 vs. PD2	114	0.04	0.815
**Less affected side/controls**										
Double Support (%)	Control (*n* = 33)	15.424	6.788	8.062	0.10	0.018 *	Control vs. PD 1–1.5	136	0.24	0.112
	PD 1–1.5 (*n* = 12)	21.778	16.864				Control vs. PD 2	150	0.38	0.008 **
	PD 2 (*n* = 17)	23.213	11.149				PD 1–1.5 vs. PD2	80	0.18	0.330
Single support (%)	Control (*n* = 32)	38.651	4.133	0.956	0.00	0.620				
	PD 1–1.5 (*n* = 12)	36.707	6.934							
	PD 2 (*n* = 20)	38.182	5.734							
Stride length (m)	Control (*n* = 33)	1.1625	0.1570	7.529	0.09	0.023 *	Control vs. PD 1–1.5	174	0.09	0.538
	PD 1–1.5 (*n* = 12)	1.1051	0.3309				Control vs. PD 2	155	0.36	0.010 **
	PD 2 (*n* = 17)	0.9766	0.2630				PD 1–1.5 vs. PD2	56	0.38	0.042
Stride time (s)	Control (*n* = 33)	0.8666	0.1678	4.245	0.04	0.120				
	PD 1–1.5 (*n* = 12)	0.9854	0.0781							
	PD 2 (*n* = 17)	0.9444	0.1669							
Walking speed (m/s)	Control (*n* = 33)	1.4038	0.2160	7.078	0.09	0.029 *	Control vs. PD 1–1.5	183	0.06	0.700
	PD 1–1.5 (*n* = 12)	1.161	0.3157				Control vs. PD 2	151	0.38	0.008 **
	PD 2 (*n* = 17)	1.1012	0.4562				PD 1–1.5 vs. PD2	65	0.30	0.101

Values are presented as median and interquartile range (IQR). * Statistical significance for the omnibus comparisons was determined using the Kruskal–Wallis test (*p* < 0.05), and the global effect size was estimated using epsilon squared (ε2). ** Post hoc pairwise comparisons were conducted using the Mann–Whitney U test. Reported *p*-values are unadjusted two-sided values and were assessed against the Bonferroni-corrected significance threshold (α = 0.0167). Effect sizes were calculated using *r* = (*Z*/√*N*), where *N* represents the combined number of observations in the two groups being compared. Bold values indicate statistically significant results.

**Table 4:** Coefficients of variation.

Parameter	Group	Median	IQR	H	ε^2^	Sig.
Step width CV (%)	Control (*n* = 36)	25.56	11.00	4.943	0.04	0.084
	PD 1–1.5 (*n* = 14)	26.12	16.00			
	PD 2 (*n* = 23)	20.40	12.00			
**More affected side/controls**						
Stride length CV (%)	Control (*n* = 27)	4.66	3.37	3.360	0.03	0.186
	PD 1–1.5 (*n* = 8)	6.55	8.55			
	PD 2 (*n* = 18)	7.29	6.08			
Stride time CV (%)	Control (*n* = 27)	4.47	3.47	4.274	0.05	0.118
	PD 1–1.5 (*n* = 8)	1.65	3.25			
	PD 2 (*n* = 18)	3.68	1.92			
Walking speed CV (%)	Control (*n* = 27)	6.06	4.34	2.820	0.02	0.244
	PD 1–1.5 (*n* = 8)	7.21	9.09			
	PD 2 (*n* = 18)	9.84	5.27			
**Less affected side/controls**						
Stride length CV (%)	Control (*n* = 27)	4.66	3.37	0.884	0.00	0.643
	PD 1–1.5 (*n* = 12)	4.14	3.53			
	PD 2 (*n* = 17)	5.89	5.45			
Stride time CV (%)	Control (*n* = 27)	4.47	3.47	1.638	0.00	0.441
	PD 1–1.5 (*n* = 12)	2.54	5.49			
	PD 2 (*n* = 17)	3.81	1.43			
Walking speed CV (%)	Control (*n* = 27)	6.06	4.34	1.712	0.00	0.425
	PD 1–1.5 (*n* = 12)	4.72	8.24			
	PD 2 (*n* = 17)	8.21	4.74			

Values are presented as median and interquartile range (IQR). * Statistical significance for omnibus comparisons was determined using the Kruskal–Wallis test (*p* < 0.05), and effect size was estimated using epsilon squared (ε^2^). Variable-specific sample sizes are reported because some outcomes were unavailable for a subset of participants.

**Table 5:** Upper body kinematics during the iTUG walking phases.

Parameter	Group	Median	IR	*H*	ε^2^	Sig.	Comparison	*U*	*r*	Sig.
Shoulder asymmetry (%)	Control (*n* = 40)	32.077	19.61	3.456	0.02	0.178				
	PD 1–1.5 (*n* = 15)	42.036	23.26							
	PD 2 (*n* = 23)	34.499	26.21							
Elbow asymmetry (%)	Control (*n* = 40)	26.846	18.30	7.559	0.07	0.023 *	Control vs. PD 1–1.5	166	0.35	0.009 **
	PD 1–1.5 (*n* = 15)	38.137	29.86				Control vs. PD 2	355	0.21	0.103
	PD 2 (*n* = 23)	33.961	16.99				PD 1–1.5 vs. PD2	138	0.17	0.303
**More affected side/controls**										
Trunk sagittal ROM (°)	Control (*n* = 40)	.6542	0.3094	10.778	0.12	0.005 *	Control vs. PD 1–1.5	297	0.01	0.955
	PD 1–1.5 (*n* = 15)	0.5328	0.5858				Control vs. PD 2	239	0.40	0.002 **
	PD 2 (*n* = 23)	0.4238	0.2631				PD 1–1.5 vs. PD2	94	0.38	0.019
Trunk frontal ROM (°)	Control (*n* = 40)	1.499	0.3634	8.030	0.08	0.018 *	Control vs. PD 1–1.5	298	0.00	0.970
	PD 1–1.5 (*n* = 15)	1.331	0.6740				Control vs. PD 2	268	0.34	0.006 **
	PD 2 (*n* = 23)	1.2044	0.2689				PD 1–1.5 vs. PD2	106	0.32	0.047
Shoulder ROM (°)	Control (*n* = 40)	21.522	16.93	6.769	0.06	0.034 *	Control vs. PD 1–1.5	182	0.30	0.026
	PD 1–1.5 (*n* = 15)	15.889	6.374				Control vs. PD 2	321	0.25	0.047
	PD 2 (*n* = 23)	16.544	9.800				PD 1–1.5 vs. PD2	161	0.05	0.731
Elbow ROM (°)	Control (*n* = 40)	30.733	18.21	17.246	0.20	<0.001 *	Control vs. PD 1–1.5	196	0.26	0.049
	PD 1–1.5 (*n* = 15)	20.308	10.78				Control vs. PD 2	185	0.49	<0.001 **
	PD 2 (*n* = 23)	14.832	14.27				PD 1–1.5 vs. PD2	102	0.34	0.035
**Less affected side/controls**										
Trunk sagittal ROM (°)	Control (*n* = 40)	0.6542	0.3094	14.245	0.16	<0.001 *	Control vs. PD 1–1.5	259	0.10	0.438
	PD 1–1.5 (*n* = 15)	0.5546	0.5152				Control vs. PD 2	225	0.42	<0.001 **
	PD 2 (*n* = 23)	0.4391	0.1985				PD 1–1.5 vs. PD2	69	0.50	0.002 **
Trunk frontal ROM (°)	Control (*n* = 40)	1.499	0.3634	8.122	0.08	0.017 *	Control vs. PD 1–1.5	269	0.08	0.558
	PD 1–1.5 (*n* = 15)	1.3217	0.6222				Control vs. PD 2	259	0.36	0.004 **
	PD 2 (*n* = 23)	1.1329	0.3801				PD 1–1.5 vs. PD2	120	0.25	0.117
Shoulder ROM (°)	Control (*n* = 40)	21.522	16.93	0.009	0.00	0.996				
	PD 1–1.5 (*n* = 15)	20.073	20.07							
	PD 2 (*n* = 23)	23.015	13.05							
Elbow ROM (°)	Control (*n* = 40)	30.733	18.21	3.783	0.02	0.151				
	PD 1–1.5 (*n* = 15)	27.745	17.74							
	PD 2 (*n* = 23)	21.088	9.034							

Values are presented as median and interquartile range (IQR). * Statistical significance was determined using the Kruskal–Wallis test (*p* < 0.05), and effect size was estimated using epsilon squared (ε^2^). ** Statistical significance for post hoc pairwise comparisons was determined using the Mann–Whitney U test with Bonferroni correction for three comparisons (α = 0.0167), and effect sizes were calculated using r (Z/√N). Bold values indicate statistically significant results. ROM is range of motion.

**Table 6:** Lower body kinematics during the iTUG walking phases.

Parameter	Group	Median	IR	*H*	ε^2^	Sig.	Comparison	*U*	*r*	Sig.
**More affected side/controls**										
Hip ROM (sagittal) (°)	Control (*n* = 38)	40.636	5.213	5.672	0.05	0.059				
	PD 1–1.5 (*n* = 14)	40.472	10.593							
	PD 2 (*n* = 23)	37.305	7.400							
Hip ROM (frontal) (°)	Control (*n* = 38)	11.600	3.282	9.429	0.10	0.009 *	Control vs. PD 1–1.5	246	0.06	0.680
	PD 1–1.5 (*n* = 14)	11.829	7.985				Control vs. PD 2	231	0.39	0.002 **
	PD 2 (*n* = 23)	9.250	2.132				PD 1–1.5 vs. PD2	100	0.31	0.056
Knee ROM (°)	Control (*n* = 38)	58.768	5.98	4.154	0.03	0.125				
	PD 1–1.5 (*n* = 14)	57.425	12.171							
	PD (*n* = 23)	55.363	8.077							
Ankle ROM (°)	Control (*n* = 38)	33.699	16.012	8.541	0.09	0.014 *	Control vs. PD 1–1.5	202	0.18	0.187
	PD 1–1.5 (*n* = 14)	29.243	11.318				Control vs. PD 2	245	0.37	0.004 **
	PD 2 (*n* = 23)	26.789	11.770				PD 1–1.5 vs. PD2	124	0.19	0.247
**Less affected side/controls**										
Hip ROM (sagittal) (°)	Control (*n* = 38)	40.636	5.213	3.765	0.02	0.152				
	PD 1–1.5 (*n* = 14)	40.066	11.631							
	PD 2 (*n* = 23)	37.471	7.416							
Hip ROM (frontal) (°)	Control (*n* = 38)	11.600	3.282	3.509	0.02	0.173				
	PD 1–1.5 (*n* = 14)	12.306	8.037							
	PD 2 (*n* = 23)	9.642	6.020							
Knee ROM (°)	Control (*n* = 38)	58.768	5.98	3.398	0.02	0.183				
	PD 1–1.5 (*n* = 14)	61.824	13.46							
	PD 2 (*n* = 23)	58.043	4.923							
Ankle ROM (°)	Control (*n* = 38)	33.699	16.012	7.291	0.07	0.026 *	Control vs. PD 1–1.5	156	0.31	0.023
	PD 1–1.5 (*n* = 14)	29.832	12.313				Control vs. PD 2	295	0.27	0.035
	PD 2 (*n* = 23)	29.001	5.782				PD 1–1.5 vs. PD2	150	0.06	0.730

Values are presented as median and interquartile range (IQR). * Statistical significance for omnibus comparisons was determined using the Kruskal–Wallis test (*p* < 0.05), and effect size was estimated using epsilon squared (ε^2^). ** Statistical significance for post hoc pairwise comparisons was determined using the Mann–Whitney U test with Bonferroni correction for three comparisons (α = 0.0167), and effect sizes were calculated using r = (Z/√N), where N represents the total number of valid observations included in each pairwise comparison. Bold values indicate statistically significant results. ROM is range of motion.

## Data Availability

The datasets generated and/or analyzed during the current study are available from the corresponding author upon reasonable request.

## Supplementary Materials

The following supporting information can be downloaded at https://www.mdpi.com/article/10.3390/s26165177/s1, Table S1: Trial-to-trial reliability of biomechanical outcomes derived from the instrumented Timed Up and Go test.

## Abbreviations

The following abbreviations are used in this manuscript:

AI	Asymmetry Index
AP	Anteroposterior
CoM	Center of Mass
CI	Confidence Interval
CV	Coefficient of Variation
FoG	Freezing of Gait
FoGQ	Freezing of Gait Questionnaire
H&Y	Hoehn and Yahr
ICC	Intraclass Correlation Coefficient
iTUG	Instrumented Timed Up and Go
IMU	Inertial Measurement Unit
LAS	Less Affected Side
MAS	More Affected Side
MDS-UPDRS III	Movement Disorder Society–Unified Parkinson’s Disease Rating Scale Part III
mH&Y	Modified Hoehn and Yahr Scale
MMSE	Mini-Mental State Examination
PD	Parkinson’s Disease
ROM	Range of Motion
SD	Standard Deviation
TUG	Timed Up and Go
