# Supplementary material for: Instrumented Timed Up and Go Analysis Identifies Biomechanical Markers Across Early Hoehn and Yahr Stages of Parkinson’s Disease
Source: Sensors (Basel). 2026 Aug 15;26(16):5177. doi: 10.3390/s26165177 (PMC13517892; doi:10.3390/s26165177)
Supplement: Supplementary file 1 [file sensors-26-05177-s001.zip › sensors-4417650-supplementary.pdf]

Supplementary Table S1. Trial-to-trial reliability of biomechanical outcomes derived from the instrumented Timed Up and Go test.

**Outcome measure**	**Side**	**Participants with at least two valid trials**	**Valid trials per participant**	***Valid trial observations, n***	***k*eff**	**ICC (1,1)**	**95% CI**	**ICC (1,*k*eff)**	**95% CI**
Total iTUG (s)	—	79	2 (n=12)<br>3 (n=7)<br>4 (n=60)	285	3.39	0.94	0.89–0.96	0.98	0.96–0.99
Sit to stand (s)	—	79	2 (n=12)<br>3 (n=7)<br>4 (n=60)	285	3.39	0.56	0.28–0.66	0.81	0.57–0.87
Walking go (s)	—	79	2 (n=12)<br>3 (n=7)<br>4 (n=60)	285	3.39	0.86	0.66–0.91	0.95	0.87–0.97
Walking return (s)	—	79	2 (n=12)<br>3 (n=7)<br>4 (n=60)	285	3.39	0.85	0.70-0.92	0.95	0.89-0.97
First turn (s)	—	79	2 (n=12)<br>3 (n=7)<br>4 (n=60)	285	3.39	0.76	0.66–0.84	0.92	0.86–0.95
Second turn (s)	—	79	2 (n=12)<br>3 (n=7)<br>4 (n=60)	285	3.39	0.78	0.67-0.83	0.92	0.87-0.94
Stand to sit (s)	—	79	2 (n=12)<br>3 (n=7)<br>4 (n=60)	285	3.39	0.73	0.57–0.80	0.90	0.81–0.93
Step width	—	73	2 (n=12)<br>3 (n=5)<br>4 (n=56)	263	3.37	0.76	0.68–0.83	0.91	0.88–0.94
Turning area	—	79	2 (n=12)<br>3 (n=4)<br>4 (n=63)	288	3.42	0.79	0.72–0.86	0.93	0.90–0.96
Double support (%)	MAS	51	2 (n=13)<br>3 (n=9)<br>4 (n=29)	169	3.04	0.80	0.69–0.87	0.93	0.86–0.96
Double support (%)	LAS	55	2 (n=13)<br>3 (n=12)<br>4 (n=30)	182	3.06	0.78	0.69–0.85	0.92	0.87–0.95
Single support (%)	MAS	56	2 (n=14)<br>3 (n=12)<br>4 (n=30)	184	3.03	0.71	0.56–0.81	0.88	0.80–0.93
Single support (%)	LAS	53	2 (n=15)<br>3 (n=8)<br>4 (n=30)	174	3.00	0.81	0.68–0.88	0.93	0.87–0.96
Stride length (m)	MAS	53	2 (n=15)<br>3 (n=8)	174	3.00	0.94	0.84–0.97	0.98	0.94–0.99

			4 (n=30)						
Stride length (m)	LAS	56	2 (n=14)<br>3 (n=12)<br>4 (n=30)	184	3.03	0.91	0.84–0.95	0.97	0.94–0.98
Stride time (s)	MAS	53	2 (n=15)<br>3 (n=8)<br>4 (n=30)	174	3.00	0.87	0.77–0.93	0.95	0.91–0.97
Stride time (s)	LAS	56	2 (n=14)<br>3 (n=12)<br>4 (n=30)	184	3.03	0.86	0.74–0.92	0.95	0.90–0.97
Walking speed (m/s)	MAS	53	2 (n=15)<br>3 (n=8)<br>4 (n=30)	174	3.00	0.94	0.88-0.97	0.98	0.96-0.99
Walking speed (m/s)	LAS	56	2 (n=14)<br>3 (n=12)<br>4 (n=30)	184	3.03	0.89	0.81-0.94	0.96	0.93-0.98
Shoulder asymmetry (%)	—	74	2 (n=11)<br>3 (n=11)<br>4 (n=52)	263	3.34	0.70	0.60–0.77	0.89	0.83–0.92
Elbow asymmetry (%)	—	74	2 (n=11)<br>3 (n=11)<br>4 (n=52)	263	3.34	0.62	0.44–0.73	0.84	0.72–0.90
Trunk sagittal ROM (°)	MAS	78	2 (n=13)<br>3 (n=6)<br>4 (n=59)	280	3.35	0.80	0.71–0.86	0.93	0.89–0.95
Trunk sagittal ROM (°)	LAS	77	2 (n=10)<br>3 (n=8)<br>4 (n=59)	280	3.43	0.61	0.34–0.85	0.84	0.64–0.95
Trunk frontal ROM (°)	MAS	78	2 (n=13)<br>3 (n=6)<br>4 (n=59)	280	3.35	0.90	0.83–0.93	0.97	0.94–0.98
Trunk frontal ROM (°)	LAS	77	2 (n=10)<br>3 (n=8)<br>4 (n=59)	280	3.43	0.85	0.80–0.89	0.95	0.93–0.96
Shoulder ROM (°)	MAS	78	2 (n=13)<br>3 (n=6)<br>4 (n=59)	280	3.35	0.89	0.83–0.92	0.96	0.94–0.97
Shoulder ROM (°)	LAS	77	2 (n=10)<br>3 (n=8)<br>4 (n=59)	280	3.43	0.88	0.81–0.91	0.96	0.94–0.97
Elbow ROM (°)	MAS	78	2 (n=13)<br>3 (n=6)<br>4 (n=59)	280	3.35	0.89	0.84–0.93	0.96	0.94–0.98
Elbow ROM (°)	LAS	77	2 (n=10)<br>3 (n=8)<br>4 (n=59)	280	3.43	0.89	0.84–0.92	0.96	0.94–0.98

Hip sagittal ROM (°)	MAS	75	2 (n=13)<br>3 (n=7)<br>4 (n=55)	267	3.32	0.85	0.77–0.90	0.95	0.92–0.97
Hip sagittal ROM (°)	LAS	77	2 (n=12)<br>3 (n=5)<br>4 (n=60)	279	3.40	0.85	0.77–0.90	0.95	0.92–0.97
Hip frontal ROM (°)	MAS	75	2 (n=13)<br>3 (n=7)<br>4 (n=55)	267	3.32	0.79	0.71–0.84	0.93	0.89–0.95
Hip frontal ROM (°)	LAS	77	2 (n=12)<br>3 (n=5)<br>4 (n=60)	279	3.40	0.84	0.78–0.89	0.95	0.92–0.96
Knee ROM (°)	MAS	75	2 (n=13)<br>3 (n=7)<br>4 (n=55)	267	3.32	0.87	0.81–0.90	0.96	0.93–0.97
Knee ROM (°)	LAS	77	2 (n=12)<br>3 (n=5)<br>4 (n=60)	279	3.40	0.91	0.87–0.94	0.97	0.96–0.98
Ankle ROM (°)	MAS	75	2 (n=13)<br>3 (n=7)<br>4 (n=55)	267	3.32	0.91	0.88–0.94	0.97	0.96–0.98
Ankle ROM (°)	LAS	77	2 (n=12)<br>3 (n=5)<br>4 (n=60)	279	3.40	0.86	0.79–0.92	0.96	0.93–0.97

Note: ICCs were estimated using a one-way random-effects, absolute-agreement model fitted by restricted maximum likelihood. ICC(1,1) represents single-trial reliability, whereas ICC(1,k_eff) represents the reliability of the participant-level mean. The effective number of valid trials (k_eff) was calculated as the harmonic mean of the participant-specific numbers of valid trials. Each outcome-specific analysis included participants with at least two valid trial-level values, and all available valid trials were retained. The 95% confidence intervals were estimated using participant-level bootstrap resampling with 2,000 samples. ICC estimates and confidence limits were rounded to two decimal places**.**

Abbreviations: CI, confidence interval; ICC, intraclass correlation coefficient; iTUG, instrumented Timed Up and Go; LAS, less affected side; MAS, more affected side; ROM, range of motion
